# Transcriptomic analysis of spleen B cell revealed the molecular basis of bursopentin on B cell differentiation

**DOI:** 10.1186/s13567-022-01123-z

**Published:** 2022-12-14

**Authors:** Ze Zhang, Jiaxi Cai, Shanshan Hao, Chenfei Li, Jiajing Chen, Tongtong Li, Xiuli Feng

**Affiliations:** 1 Key Laboratory of Animal Microbiology of China’s Ministry of Agriculture, College of Veterinary Medicine, Nanjing Agricultural University, Nanjing, 210095 China; 2 MOE Joint International Research Laboratory of Animal Health and Food Safety, College of Veterinary Medicine, Nanjing Agricultural University, Nanjing, 210095 China

**Keywords:** BP5, B cell differentiation, enrichment pathways, transcriptional factor, immune induction

## Abstract

The bursa of Fabricius, the acknowledged humoral immune organ unique to birds, plays a vital role in B cell development. Bursopentin (BP5) derived from the bursa is reported to induce the development and formation of B cells. However, the mechanism of BP5 on B cell differentiation is still unclear. In this paper, total B lymphocytes from mice immunized with H9N2 subtype AIV vaccine were stimulated with BP5. The results show that BP5 at the experimental dosages promoted B cell differentiation, including the total B cells, activated B cells, differentiated B cells, mature B cells and plasma cells. Then, the in vivo immune experiment proved that the percentages of activated and differentiated B cells from mice immunized with AIV vaccine and 0.25 mg/mL BP5 were increased. To investigate the molecular mechanism of BP5 on B cell differentiation, the gene expression profiles of B cells purified from the spleen cells of mice immunized with AIV vaccine and BP5 were detected following RNA sequencing technology. The results show that BP5 at 0.05 and 0.25 mg/mL induced the enrichment of various biological functions, and stimulated five common significant enrichment pathways in B cells from the immunized mice. Additionally, 120 and 59 differentially expressed genes (DEG) represented transcriptional factors in B cells following 0.05 and 0.25 mg/mL BP5 immunization, respectively. In summary, these results suggest that BP5 regulates various gene expression involved in regulation of B cell development, which provides the knowledge required for additional studies on B cell differentiation in response to bursal-derived peptides and also provides an important experimental basis for improving vaccine immunity.

**Supplementary Information:**

The online version contains supplementary material available at 10.1186/s13567-022-01123-z.

## Introduction

The bursa of Fabricius (BF) is a lymphoid organ only existing in birds, which is vital to B cell development and antibody production [1]. During the embryonic development, stem cells in the hematopoietic system gradually differentiate into complete lymphocytes in BF, accompanied with a variety of regulatory factor signals [2, 3]. As the function of BF as the central humoral immune organ has gradually been determined, more and more research is focused on improving the understanding of the active components in BF enhancing immune response.

Bursin is the first active factor with a clear tripeptide structure Lys-His-Gly-NH2 derived from BF, and selectively induces B-cell-differentiation [4, 5]. Bursin-like peptide (BLP) is reported to have the potential as a virus inhibitor and immunity regulator to decrease the negative effects of ALV infection in SPF chickens [6]. BP-IV induces a strong humoural and cellular immune response for the epitope peptide vaccine for H9N2 subtype AIV [7]. Bursal peptides BP9 and BP7 induce a significant antibody response and B cell autophagy [8, 9], and also stimulate a number of differentially expressed genes in avian immature B cells [10]. Furthermore, bursal hexapeptide (KGNRVY) and pentapeptide (MPPTH) are reported to effectively induce the AIV-specific antibody, T cell and antigen presentation immune responses [11]. Bursal hexapeptide (BHP, AGCCNG) and bursal septpeptide II (BSP-II) are reported to inhibit tumor cell proliferation via the p53 signaling pathway [12, 13]. Therefore, the active peptides derived from BF have a variety of biological functions on immune responses.

BP5 with Cys-Lys-Arg-Val-Tyr was recently identified as an active peptide from chicken bursa extract [14], which markedly promotes B cell development by increasing CFU-pre B [15], and plays a role in the inhibition of oxidative stress response and GSH redox cycle regulation [16]. Additionally, BP5 significantly decreases the pro-inflammatory factors IL-1β, IL-6, TFN-α and anti-inflammatory factors in LPS induced dendritic cells [17]. The molecular basis of transcriptomes of BP5 on B lymphocyte differentiation is of great significance since BF plays a vital role in controlling B-cell development to differentiation [18, 19].

In this study, we detected the inducing roles of BP5 on B cell differentiation in vivo and in vitro, and investigated the molecular basis of BP5 on B cell differentiation using RNA sequencing technology. Also, the enriched biological functions, pathways and differentially expressed transcription factors on the differentiated B cells with BP5 treatment were further analyzed. The study provides novel insight into the B cell differentiation mechanism, and provides the theoretical support for vaccine adjuvant development.

## Materials and methods

### Peptides, AIV vaccine and mice

BP5 was synthesized by GenScript Biotechnology Co., Ltd (Nanjing, China), and the purity determined by HPLC and mass spectrometry was 98.8%. Avian influenza virus strain A / chicken/Shandong/Ly1/2017(H9N2) was stored in our laboratory [20]. Virus was harvested from the allantoic fluid of 9-day chicken embryos, and the hemagglutination titer of the virus was 2^9^. After inactivation, virus antigen was prepared for the experimental vaccine with adjuvant ISA206.

6-week-old SPF ICR mice were purchased from the experimental animal center of Yangzhou University (Yangzhou, China).

### In vitro stimulation and detection

#### Mice immunization and antibody detection

Twenty 6-week-old SPF ICR mice were divided into a vaccine group and control group, with 10 mice in each group. Mice in the vaccine group were triple injected intraperitoneally at 2-week intervals with the experimental vaccine. On the 14th day after third immunization, serum samples were collected from the immunized mice to detect the specific IgG antibody production with indirect ELISA methods established with 2 μg/mL AIV antigen coated plate [9]. Also, HI antibody levels in the immunized mice were detected based on 4 HA, according to the established method [21].

#### Spleen B cell isolation

The spleen B cells were isolated from the immunized mice on the 7^th^ day after third immunization, and purified with magnetic beads, and treated with 1, 0.1, and 0.01 µg/mL BP5 for 48 h, in which the cells were treated with 1 ng/mL BSA as the unrelated polypeptide control, and LPS as a positive control for B cell differentiation induction. After MTT incubation for 4 h, 100 µL/well DMSO was added to lyse the cells, and the OD values were detected at 570 nm wavelength, and were analyzed according to the following formula to detect the spleen cell viabilities.

$${\text{Stimulation index }}\left( {{\text{SI}}} \right) = \frac{{{\text{OD }}\left( {\text{experimental group}} \right)}}{{{\text{OD }}\left( {\text{unrelated polypeptide control}} \right)}}.$$

#### Flow cytometry

B cells isolated from the spleen treated with BP5 were incubated with fluorescein labeled antibodies CD19-FITC, CD69-PerCP-Cy5.5, CD43-PE, CD38-Alexa Flour 647, CD27-PE and IgD-APC for 30 min, respectively. After washing twice, the incubated cells were resuspended in 500 μL PBS buffer and analyzed by flow cytometry (BECKMAN COULTER, cytoflex).

### In vivo stimulation and detection

Forty 6-week-old SPF ICR mice were divided into four experimental groups with 10 mice in each group, including PBS, vaccine plus 10 µg/mL BSA, vaccine plus 0.25 mg/mL BP5 and vaccine plus 0.05 mg/mL BP5. Mice were injected intraperitoneally twice at two-week intervals, respectively. On 7^th^ day after the second immunization, the spleen cells collected from the experimental group were incubated with fluorescein labeled antibodies to detect different B cell subtypes in vivo. Also, two weeks after the second immunization, serum samples were collected from the immunized mice to detect specific antibody production.

#### B cell preparation, library establishment, sequencing and transcriptome analysis

Spleen B cells were isolated from mice immunized with vaccine, vaccine plus 0.05 mg/mL BP5 and vaccine plus 0.25 mg/mL BP5, respectively, and were purified according to B220 magnetic bead Kit instructions. The transcriptome analysis was accomplished in the Tsingke Biotechnology Company (Beijing, China). Briefly, the total RNA were extracted with Trizol from each group, and the concentration, purity and integrity of the total RNA sample were detected by quantitative agar gel electrophoresis. The mRNA were enriched with oligo (dT) magnetic beads from the RNA samples, and then were fragmented, and reverse transcribed with the random primers. After terminal repair and addition of specific adaptors with poly A tailing, the samples were then analyzed by sequencing.

The expression level of each transcript was calculated according to the number of fragments per thousand base pairs per million exons. Additionally, based on gene ontology (GO) and Kyoto encyclopedia of genes and genomes (KEGG) pathway database, the DEGs were compared and analyzed with the thresholds of *p* value less than 0.05.

### Fluorescence quantitative PCR

The total RNA were isolated from three experimental groups, and were reverse transcribed with PrimeScript™ RT Master Mix according to the procedure at 37 ℃ for 15 min and 85 ℃ for 5 s. The cDNA obtained were used as a template for fluorescence quantitative PCR (639676, Takara). BC018473, St6galnac1, Tmod2, Bmp8b, Tnfrsf21, Csf1r and Ackr3 seven DEG were detected with fluorescence qRT-PCR analysis, and β-actin was used as the internal reference control. The primers of the seven selected genes are listed in Additional file 1. The data were calculated and the gene expression levels were analyzed with the 2^−△△CT^ method [22]. QPCR data were repeated three times independently, and the experimental result diagram was analyzed by GraphPad Prism 6.01 statistical software.

## Results

### Establishment of the immune B cell model in vitro

The serum samples of immunized mice were collected at 2 weeks after the third immunization to detect the antibody levels by indirect ELISA and HI methods. As shown in Additional file 2A, the specific IgG antibody levels in mice immunized with AIV vaccine after three immunizations were significantly higher than those in the PBS control. Also, HI antibodies produced from mice immunized with AIV vaccine were significantly higher than those of PBS control (Additional file 2B). B lymphocytes were isolated with magnetic beads from the spleen cells of the mice immunized with H9N2 AIV inactivated vaccine. As shown in Additional file 2C, the purity of B lymphocytes was 95.2%. Furthermore, BP5 from 0.01 to 1 µg/mL could stimulate B lymphocyte proliferation activity, in which the cell viabilities from 0.01 µg/mL BP5 treatment were the highest (Additional file 2D). The results prove the establishment of the immune B cell model in vitro.

### BP5 promotes the proportion of total B cell in vitro

**Figure 1:**
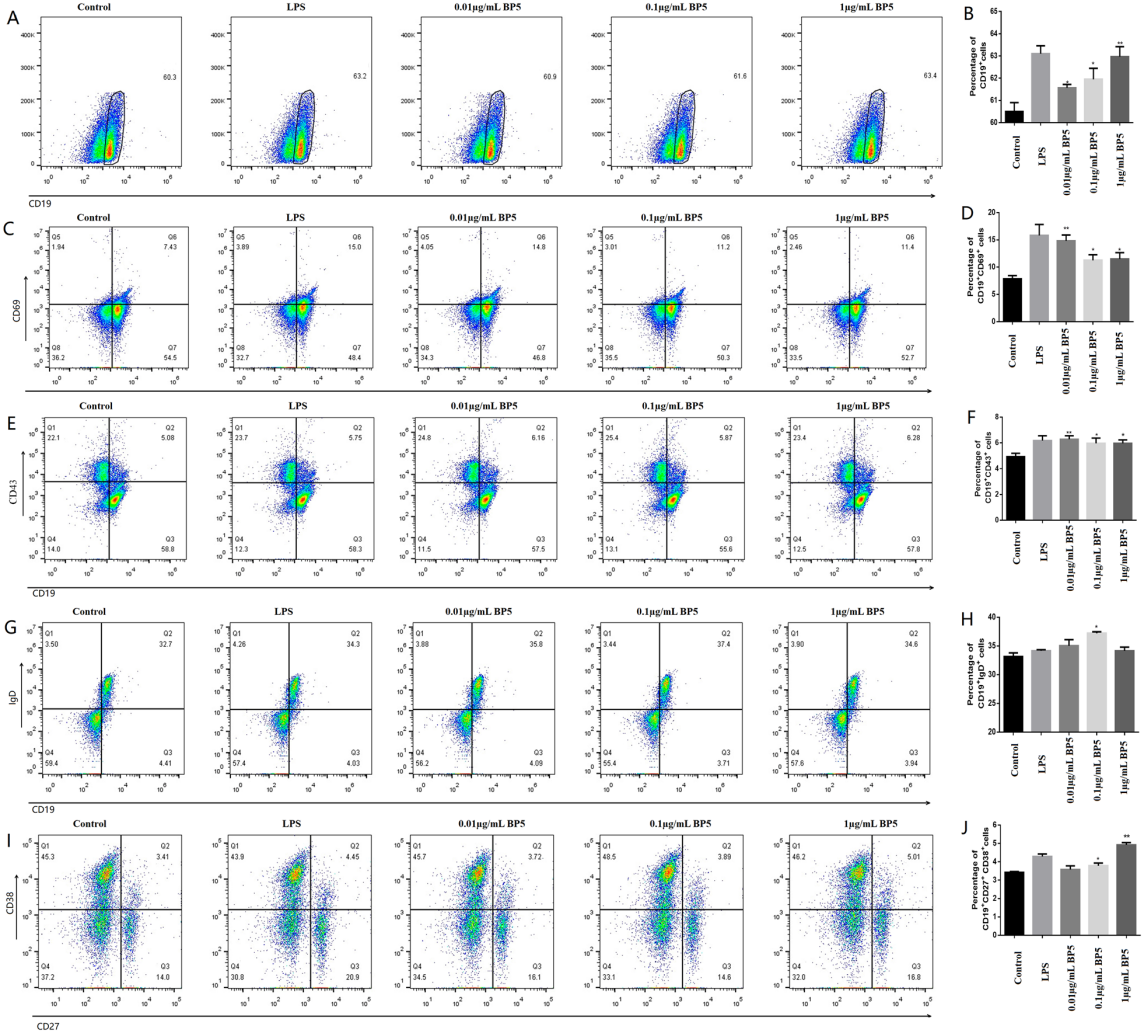
**BP5 induced B cell differentiation in vitro. A** Flow cytometry plot of CD19^+^ B cells with BP5 treatment. **B** Histogram of CD19^+^ B cell percentages with BP5 treatment. **C** Flow cytometry plot of CD19^+^CD69^+^ B cells. **D** Histogram of CD19^+^CD69^+^ B cell percentages with BP5 treatment. **E** Flow cytometry plot of CD19^+^CD43^+^ B cells with BP5 treatment. **F** Histogram of CD19^+^CD43^+^ B cell percentages with BP5 treatment. **G** Flow cytometry plot of CD19^+^IgD^+^ B cells with BP5 treatment. **H** Histogram of CD19^+^IgD^+^ B cell percentages with BP5 treatment. **I** Flow cytometry plot of CD19^+^CD27^+^CD38^+^ B cells with BP5 treatment. **J** Histogram of CD19^+^CD27^+^CD38^+^ B cell percentages with BP5 treatment. Data represent the mean ± standard deviation (S.D.). Significant differences between groups were determined using the student *t*-test. *P < 0.05, **P < 0.01.

In order to explore the effect of BP5 on the differentiation of total B cells in vitro, spleen B lymphocytes from the immunized mice with H9N2 AIV inactivated vaccine were treated with BP5 at 0.01, 0.1 and 1 µg/mL for 48 h. The results show that compared with the control group, the experimental concentrations of BP5 could promote the proportion of CD19^+^total B cells, and the amounts of CD19^+^total B cells were increased with an increase of BP5 concentration. It was observed that the proportion of CD19^+^ total B cells treated with 1 µg/mL BP5 was the highest among the experimental groups, which was similar to that of the LPS control (Figures 1A and B). These results indicate that BP5 could stimulate the proliferation and differentiation of total B cells.

### BP5 induces B cell activation in vitro

In order to study the effect of BP5 on B cell activation in vitro, B lymphocytes treated with BP5 were incubated with labeled monoclonal antibodies specific to CD19 and CD69. FCM results show that the percentages of CD19^+^CD69^+^ activated B cells treated with BP5 were significantly increased, compared with that of the control, in which 0.01 µg/mL BP5 stimulated the highest percentages of activated B cells among three concentrations of BP5 (Figures 1C and D). These results show that BP5 could promote B cell activation, but the relationship between dosages and B cell activation needs to be further explored.

### BP5 promotes B cell differentiation in vitro

To explore the effect of BP5 on the differentiation of B cells in vitro, the proportions of CD19^+^CD43^+^ differentiated B cells in B lymphocytes treated with BP5 were detected. The results show that compared with the control, BP5 at 0.01, 0.1 and 1 µg/mL significantly promoted B cell differentiation (Figures 1E and F), which were similar to that of LPS control, indicating that BP5 could stimulate B cell differentiation.

### BP5 induces B cell maturation in vitro

In order to observe the effect of BP5 on the development process of B cells in vitro, we also detected the function of BP5 on mature B cells in B lymphocytes. The results show that the populations of CD19^+^IgD^+^ B cells treated with three concentrations of BP5 were significantly higher than those of the control (Figures 1G and H), in which the proportions of CD19^+^IgD^+^ B cells stimulated with 0.1 µg/mL BP5 were the highest. These results suggest that BP5 could regulate B cell maturation.

### BP5 promotes the production of plasma cells in vitro

To investigate the function of BP5 on plasma cells in vitro, we also detected the proportions of CD19^+^CD27^+^CD38^+^ plasma cells (Figures 1I and J). Compared with the control, when the BP5 concentration was increased to 1 µg/mL, the plasma cell populations were significantly increased, which was also higher than that of the LPS control. The results show that BP5 could promote the production of plasma cells.

### BP5 induces B cell activation in vivo

**Figure 2:**
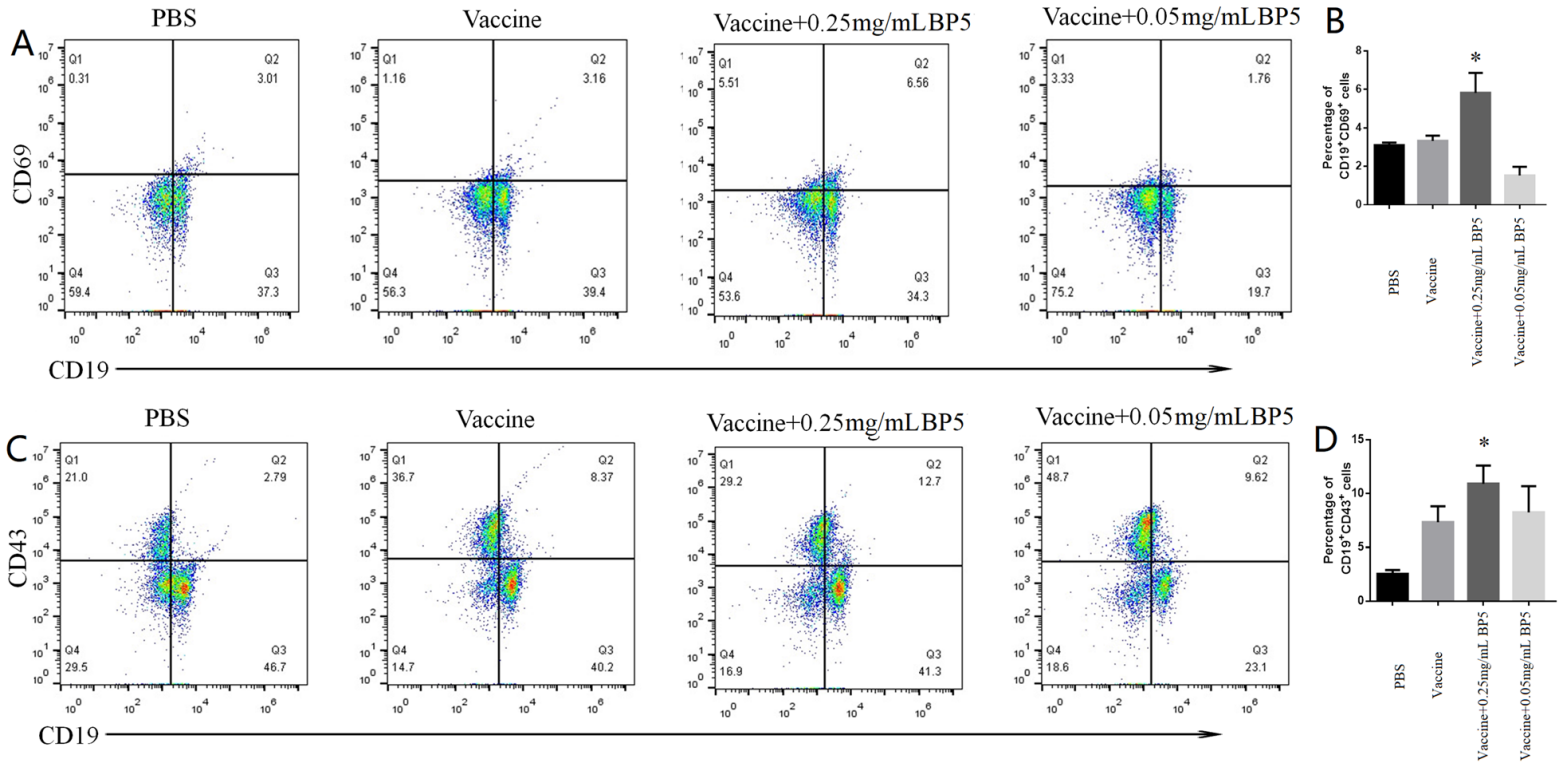
**BP5 induced B cell differentiation in vivo. A** Flow cytometry plot of CD19^+^CD69^+^ B cells from mice immunized with BP5 and AIV vaccine. **B** Histogram of CD19^+^CD69^+^ B cells from mice immunized with BP5 and AIV vaccine. **C** Flow cytometry plot of CD19^+^CD43^+^ B cells from mice immunized with BP5 and AIV vaccine. **D** Histogram of CD19^+^CD43^+^ B cells from mice immunized with BP5 and AIV vaccine. Data represent the mean ± S.D. Significant differences between groups were determined using the student *t*-test. * *P* < 0.05.

To study the effects of BP5 on B cell differentiation in vivo, mice immunized with H9N2 vaccine combined with BP5 were used as the immunization model. The results show that the proportions of CD19^+^CD69^+^ activated B cells were increased in mice immunized with vaccine and 0.25 mg/mL BP5, compared with vaccine control (Figures 2A and B). Unexpectedly, the proliferation of CD19^+^CD69^+^ activated B cells decreased in the 0.05 mg/mL combined vaccine group (Figures 2A and B). The dose relationship between BP5 and B cell activation needs to be further studied.

### BP5 induces B cell differentiation in vivo

Also, the proportions of CD19^+^CD43^+^ differentiated B cells in spleen cells collected from mice immunized with vaccine plus 0.05 or 0.25 mg/mL BP5 were higher than those of the vaccine control (Figures 2C and D). We observed that the proportions of differentiated B cells from 0.25 mg/mL BP5 immunization were the highest among all the experimental groups.

### Sequencing library analysis

Three sequencing libraries were prepared from spleen B cell samples obtained from vaccine control, 0.05 and 0.25 mg/mL BP5 to analyze the gene expression profiles using transcriptome sequencing.

The data was refined by discarding low-quality reads, Ns Reads, Adapter Polluted Reads and Raw Q30 Bases. As shown in Table 1, in total, 41.17 million high-quality reads were generated from the control, 41.03 million for 0.05 mg/mL BP5 and 40.72 million for 0.25 mg/mL BP5 samples, respectively. The mapped reads were 39.48, 39.49 and 39.26 million for the control, 0.05 and 0.25 mg/mL BP5, in which the mapping rates were 95.87%, 96.23% and 96.41% for the control, 0.05 and 0.25 mg/mL BP5, respectively. Additionally, multiMap reads were 2.25, 2.38 and 2.21 million for the control, 0.05 and 0.25 mg/mL BP5, in which the multiMap rates were 5.46%, 5.8% and 5.42% for control, 0.05 and 0.25 mg/mL BP5, respectively.

**Table 1:** **Comparison rate analysis among three experimental groups**

Library	Control	0.05 mg/mL BP5	0.25 mg/mL BP5
Total Reads	41 176 790	41 033 966	40 718 632
Mapped Reads	39 476 687	39 485 765	39 257 436
Mapping Rate	0.9587	0.9623	0.9641
UnMapped Reads	1 700 103	1 548 201	1 461 196
MultiMap Reads	2 248 561	2 379 316	2 205 097
MultiMap Rate	0.0546	0.058	0.0542

**Figure 3:**
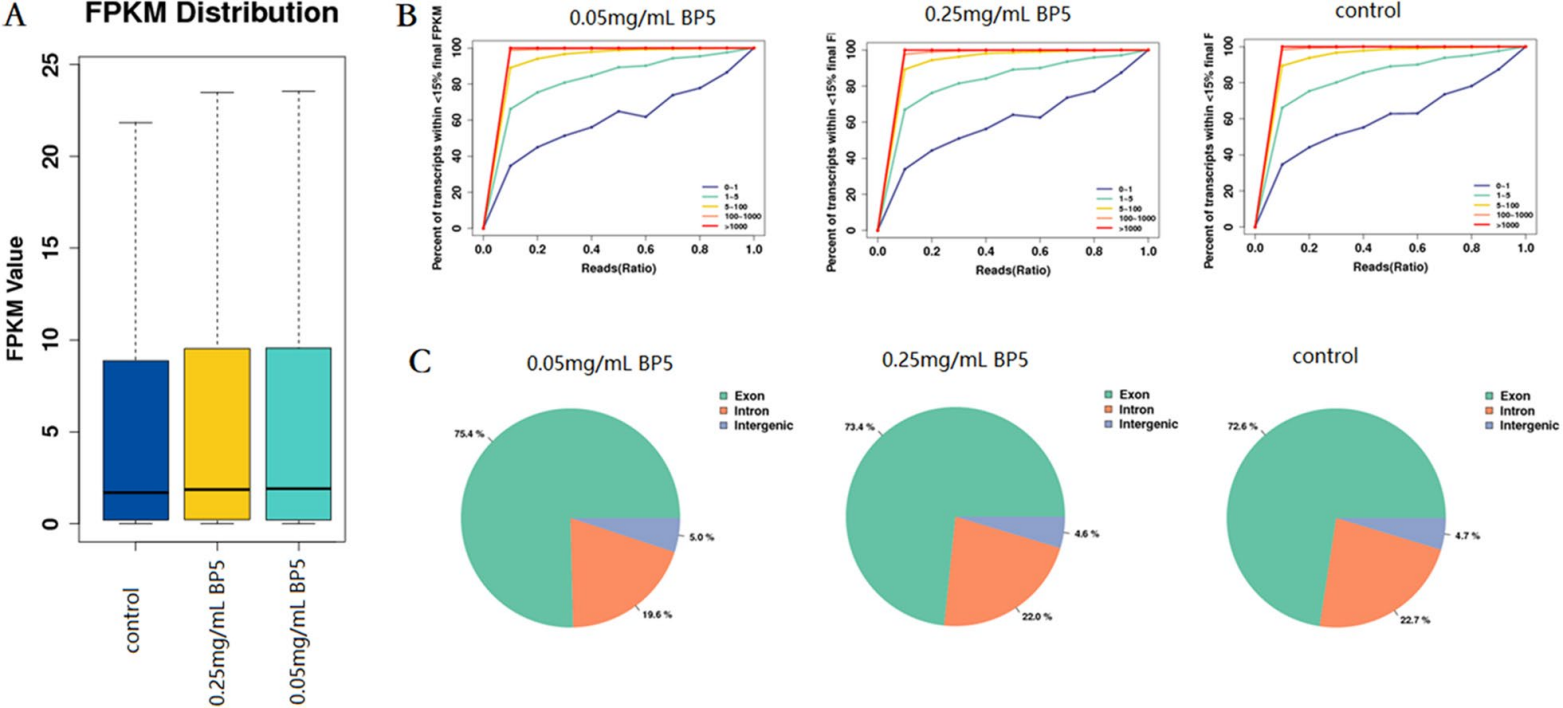
**Mapping analysis of three experimental groups.** RNA sequencing was employed to analyze all mRNA expression of spleen B cells from mice immunized with or without BP5. **A** Distribution. The box chart showed the distribution of gene expression levels of three samples. **B** Saturation. Different colors represented genes classified according to the level of expression. According to the depth of each saturation curve reaching the platform period, the data volume of these experiments reached the needs of further data analysis. **C** Mapping region. According to the sequence alignment information, the number of sequences aligned to exons, introns and intergenic regions were counted, respectively; a pie chart indicating the proportions was made.

Furthermore, the expression of three samples were determined with a logarithm based on 2 for the gene expressions of the three experimental samples, and then the density distribution maps were made. The box diagrams show the distribution of the transcript levels of three groups (Figure 3A). The saturations of gene expression from three groups were shown in Figure 3B. The mapping regions of three groups were listed in Figure 3C and Additional file 3, in which the exon, intron and intergenic regions for the control were 72.56%, 22.75% and 4.69%, and for 0.05 mg/mL BP5 was 75.38%, 19.59% and 5.01%, and for 0.25 mg/mL BP5 was 73.36%, 22.00% and 4.64%, respectively. These results suggest that three sequencing libraries were of sufficient quality.

### Comparative analysis of gene expression profiles in B cell response to BP5

**Figure 4:**
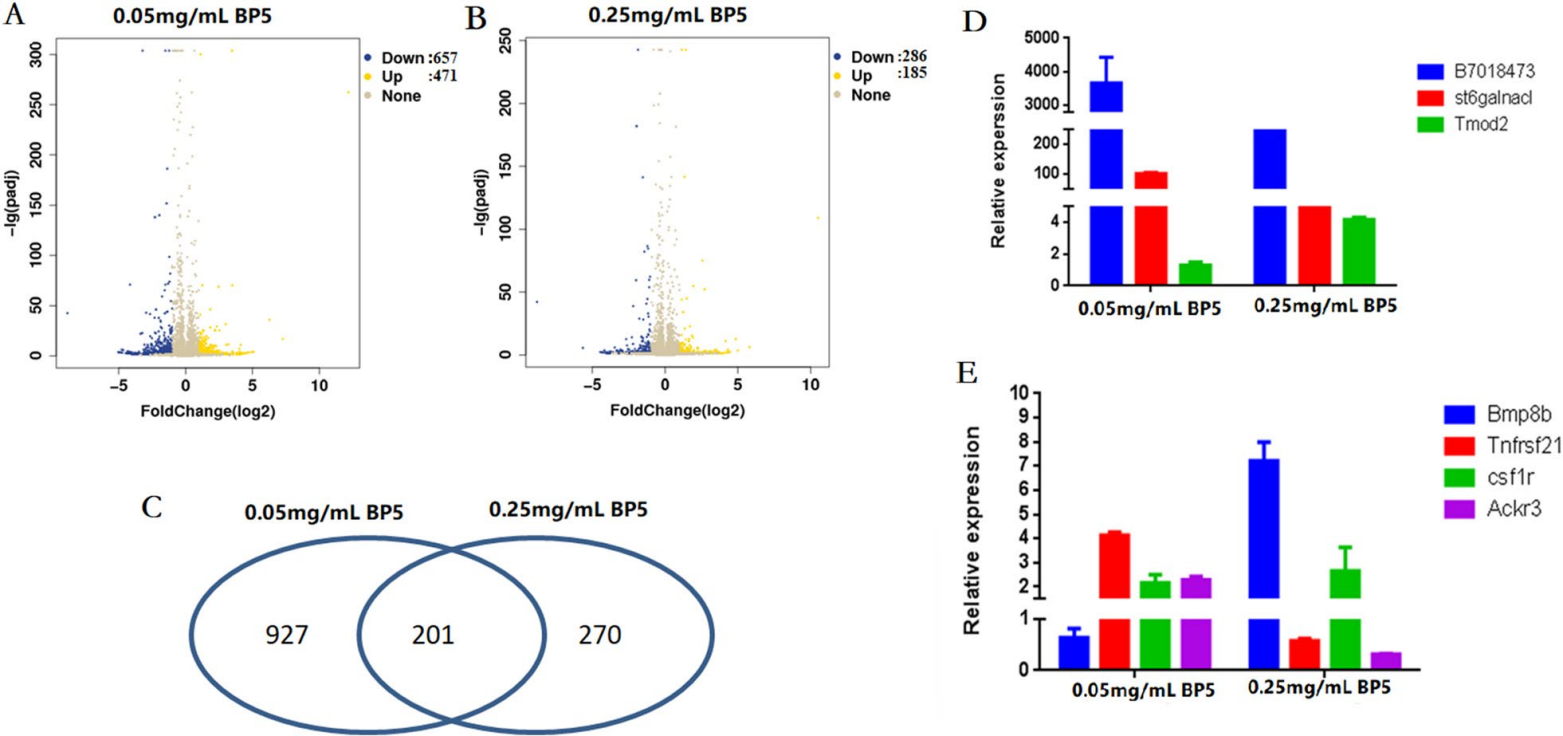
**Differential gene expression profiling and verification of B cells immunized with 0.05 and 0.25 mg/mL BP5.** Volcano plots and venn diagram of DEG in B cells from the spleen cells of mice immunized with or without BP5. **A** Volcano plot of DEG in response to 0.05 mg/mL BP5. Each dot was a gene. Blue dots: down-regulated, *P* value < 0.05 and log_2_ Fold Change ≥ 1.5, Orange dots: up-regulated, *P* value < 0.05 and log_2_ Fold Change ≥ 1.5. Grey dots did not fit these criteria. **B** Volcano plot of DEG in response to 0.25 mg/mL BP5. Each dot was a gene. Blue dots: down-regulated, *P* value < 0.05 and log_2_ Fold Change ≥ 1.5, Orange dots: up-regulated, *P* value < 0.05 and log_2_ Fold Change ≥ 1.5. Grey dots did not fit these criteria. **C** Venn diagram showing the number of DEG between 0.05 and 0.25 mg/mL BP5. These genes were shared between both comparisons. **D-E** Fold-change expression of related genes in B cells from mice spleen immunized with BP5. qRT-PCR was performed for each gene to validate the RNA-Seq results. Seven genes were selected from the DEG results from the comparison of 0.05 and 0.25 mg/mL BP5, with FDR 0.05 and log_2_ fold change cut-off. The housekeeping gene β-actin was used to normalize relative expression. Statistically significant differences were determined using the student *t*-test.

In this experiment, the gene expression profiles of B cells from mice immunized with H9N2 vaccine together with 0.05 and 0.25 mg/mL BP5 were studied with transcriptome sequencing. DEG had changes in expression intensity by over two times (fold ≥ 2 and *P* value < 0.05) in the treatment group as compared to that of the vaccine control as shown in Figure 4.

The microarray results show that there were 1128 DEG in spleen B cells from mice following 0.05 mg/mL BP5 immunization, including 471 upregulated and 657 downregulated genes (Figure 4A). Upon 0.25 mg/mL BP5 immunization, there were 471 DEG, including 185 upregulated and 286 downregulated genes (Figure 4B). It was observed that 201 DEG commonly appeared in both 0.05 and 0.25 mg/mL immunizations (Figure 4C). There were 927 specifically regulated genes following 0.05 mg/mL BP5 immunization, and 270 specifically regulated genes upon 0.25 mg/mL BP5 immunization.

### Validation of DEG by qRT-PCR

To verify the accuracy and reliability of DEG from microarray analysis, in this experiment, seven DEG were selected for fluorescence qRT-PCR analysis. The results show that B7018473, St6galnadcl, Tnfrsf21, Csf1r and Ackr3 five genes were upregulated, and the expression levels of Tmod2 and Bmp8b were downregulated in 0.05 mg/mL BP5-treated B cells (Figures 4D and E). Also, B7018473, St6galnadcl, Tmod2, Bmp8b and Csf1r were upregulated, and Tnfrsf21 and Ackr3 were downregulated in 0.25 mg/mL BP5-treated B cells (Figures 4D and E). Unexpectedly, Tmod2 and Bmp8b were upregulated in 0.05 mg/mL BP5-treated B cells in microarrays, and Bmp8b and Csf1r were downregulated in 0.25 mg/mL BP5-treated B cells in microarrays (Table 2), which were dissimilar to the results of qPCR. These results indicate that the microarray data were biologically reproducible, and the gene chip analysis results were reliable.

**Table 2:** **Gene expressions of the selected seven genes in microarray**

Gene	Log_2_FoldChange (0.05 mg/mL BP5 VS Control)	Log_2_FoldChange (0.25 mg/mL BP5 VS Control)
B7018473	12.16527218	10.50851003
St6galnac1	6.271304086	4.850791183
Tmod2	4.677123056	4.314738283
Bmp8b	3.561645838	−6.028179653
Tnfrsf21	1.575721023	−3.480709705
Csf1r	1.281537919	−1.961976302
Ackr3	1.042772528	−6.028179653

Also, we observed that the relative expression levels of the same gene in B cells from 0.05 mg/mL BP5 immunization were different between microarray and qPCR results (Table 2, Figures 4D and E). For Tnfrsf21, Csf1r and Ackr3, the results obtained using the two methods were very similar, whereas the fold changes of the other four regulated genes were different. Simultaneously, expressions of St6galnadcl, Tmod2, Tnfrsf21 and Ackr3 four genes in B cells from 0.25 mg/mL BP5 immunization were similar, whereas B7018473, Bmp8b and Csf1r three gene expression were different between microarray and qPCR results. These differentiations might be related to the fluorescence signal saturation phenomenon in gene microarray hybridization, which could lead to the sensitivity of the microarray assay being lower than that of the fluorescence qRT-PCR.

### GO function analysis of DEG in response to 0.05 and 0.25 mg/mL BP5 immunization

To investigate the biological functions of BP5, the enrichment of DEG in B cells from mice immunized with vaccine and 0.05 or 0.25 mg/mL BP5in biological process, molecular function, and cellular component were analyzed, respectively.

**Figure 5:**
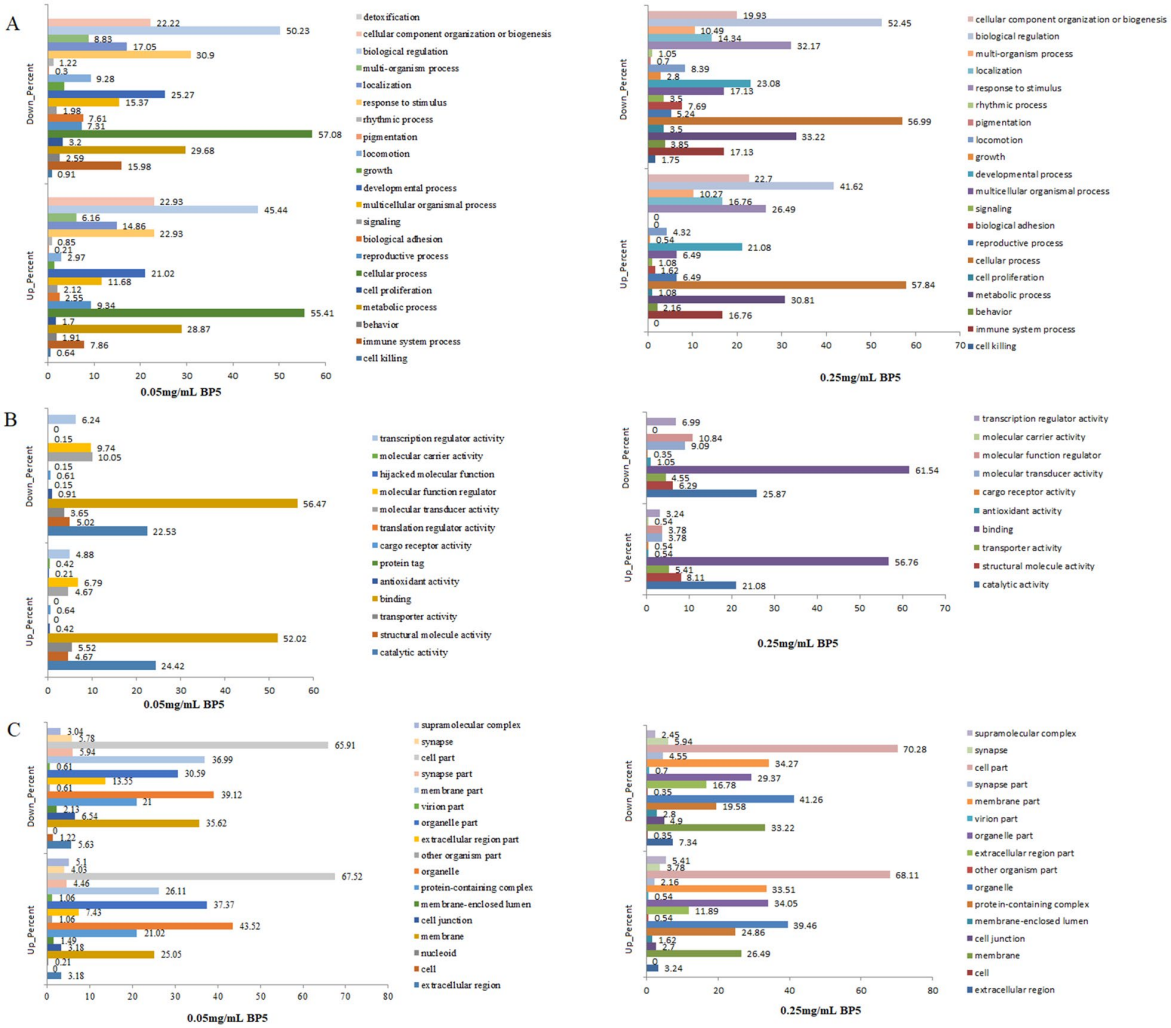
**GO enrichment analysis of DEG in spleen B cells with 0.05 and 0.25 mg/mL BP5 immunization.** Percentage by category (molecular function, biological process, and cellular component) GO terms in B cells from mice spleen immunized with 0.05 mg/mL BP5 v/s 0.25 mg/mL BP5, with *P* value < 0.05. **A **A comparative analysis of biological process between 0.05 and 0.25 mg/mL BP5 immunization. **B **A comparative analysis of molecular function between 0.05 and 0.25 mg/mL BP5 immunization. **C **A comparative analysis of cellular component between 0.05 and 0.25 mg/mL BP5 immunization.

For the up and downregulated DEG in response to both 0.05 mg/mL and 0.25 mg/mL BP5 immunizations, the significant enrichment of the biological processes were found in metabolic and cellular processes, and biological regulation (Figure 5A). For upregulated DEG in B cells with 0.05 mg/mL and 0.25 mg/mL BP5 immunizations, response to stimulus was significantly enriched response to 0.25 mg/mL BP5 immunization and relatively enriched response to 0.05 mg/mL BP5 treatment. Also, as for the downregulated DEG, the developmental process had a significantly enriched response to 0.05 mg/mL BP5 immunization and a relatively enriched response to 0.25 mg/mL BP5 immunization, and detoxification was significantly enriched only in B cells from mice immunized with 0.05 mg/mL BP5 and vaccine (Figure 5A). These results indicate that BP5 might induce various metabolic and biological processes, resulting in transcriptional regulation of B differentiation.

At the molecular function level, catalytic activity and binding were significantly enriched in response to both 0.05 and 0.25 mg/mL BP5 immunizations (Figure 5B). For the upregulated DEG in response to 0.05 and 0.25 mg/mL BP5 immunization, hijacked molecular function had a significantly enriched response to only 0.05 mg/mL BP5 immunization. Also, for downregulated genes in response to both 0.05 and 0.25 mg/mL BP5 immunization, protein tag, translation regulator activity and hijacked molecular function were only significantly enriched in B cells with 0.05 mg/mL BP5 immunization. These results indicate that BP5 could regulate the binding and catalytic activity of spleen B cells, and through regulation of expression of other molecular functions, thus responding to BP5 stimulation.

At the cellular component level, the organelle, organelle part, cell part, membrane and membrane part were significantly enriched in response to both 0.05 and 0.25 mg/mL BP5 immunization (Figure 5C). Additionally, nucleoid was significantly enriched only in 0.05 mg/mL BP5 immunization.

### KEGG analysis of Common DEG in response to 0.05 and 0.25 mg/mL BP5 immunization

To further investigate the pathway mechanism of BP5 on B cell differentiation, the distributions of DEG in pathways in B cells from mice immunized with 0.05 and 0.25 mg/mL BP5 were analyzed. Firstly, five significant enrichment pathways were observed in B cell response to 0.05 and 0.25 mg/mL BP5 immunization (Table 3), including natural killer cell mediated cytotoxicity, cytokine-cytokine receptor interaction, antigen processing and presentation, hematopoietic cell lineage, complement and coagulation cascades, which were related to B cell differentiation and development.

**Table 3:** **KEGG analysis of common DEG in response to 0.05 and 0.25 mg/mL BP5 immunization**

Name	Map	0.25 mg/mL BP5		0.05 mg/mL BP5		Co-Downregulated gene
		Up	Down	Up	Down	
Natural killer cell mediated cytotoxicity	map04650	1	9	1	10	Gzmb;Tnf;Klrk1;Klrd1;Prf1;Klre1;Ifng
Cytokine-cytokine receptor interaction	map04060	3	11	4	27	Ccl4;Tnf;Il18rap;Il1r2;Xcl1;Il1b;Tnfrsf9;Ifng;Il2rb
Antigen processing and presentation	map04612	0	8	0	12	Tnf;Klrd1;Klre1;Cd8b1;Ifng;Gm9144;Hspa1b
Hematopoietic cell lineage	map04640	1	5	1	11	Kit;Tnf;Il1r2;Il1b;Cd8b1
Complement and coagulation cascades	map04610	0	6	3	5	Plau;C3;Itgax

Furthermore, to determine the possible implications of transcripts associated with bursal peptides, the biological functions correlative to common enriched pathway analysis in enriched GO terms were analyzed in B cells from mice immunized with 0.05 and 0.25 mg/mL BP5. It was observed that various common DEG in both 0.05 and 0.25 mg/mL BP5 immunizations were involved in natural killer cell related biological processes, as shown in Additional file 4, which includes regulation of natural killer cell chemotaxis, regulation of natural killer cell mediated cytotoxicity, and natural killer cell mediated immunity. Also, some DEG participated in cytokine related biological processes (Additional file 5), including cytokine production, chemokine biosynthetic processes, interferon-gamma production, regulation of interleukin-18 and interleukin-6. Additionally, it was found that some common DEG were involved in antigen related biological and molecular function, such as antigen receptor-mediated signaling pathway, MHC class Ib receptor activity and antigen binding, shown in Additional file 6. Moreover, various common DEG in both 0.05 and 0.25 mg/mL BP5 treatment were involved in B cell and immunoglobulin related biological processes (Additional file 5), including the regulation of B cell mediated immunity and regulation of immunoglobulin mediated immune response.

In addition, 13 significant pathway enrichments were found only in B cells from mice immunized with 0.05 mg/mL BP5 (Additional file 7), and three significant pathway enrichments were found only with 0.25 mg/mL BP5 immunization (Additional file 8), in which various significant enriched pathways were found to be related to immune response functions.

### Transcriptional factor analysis of DEG in response to 0.05 and 0.25 mg/mL BP5 immunization

**Figure 6:**
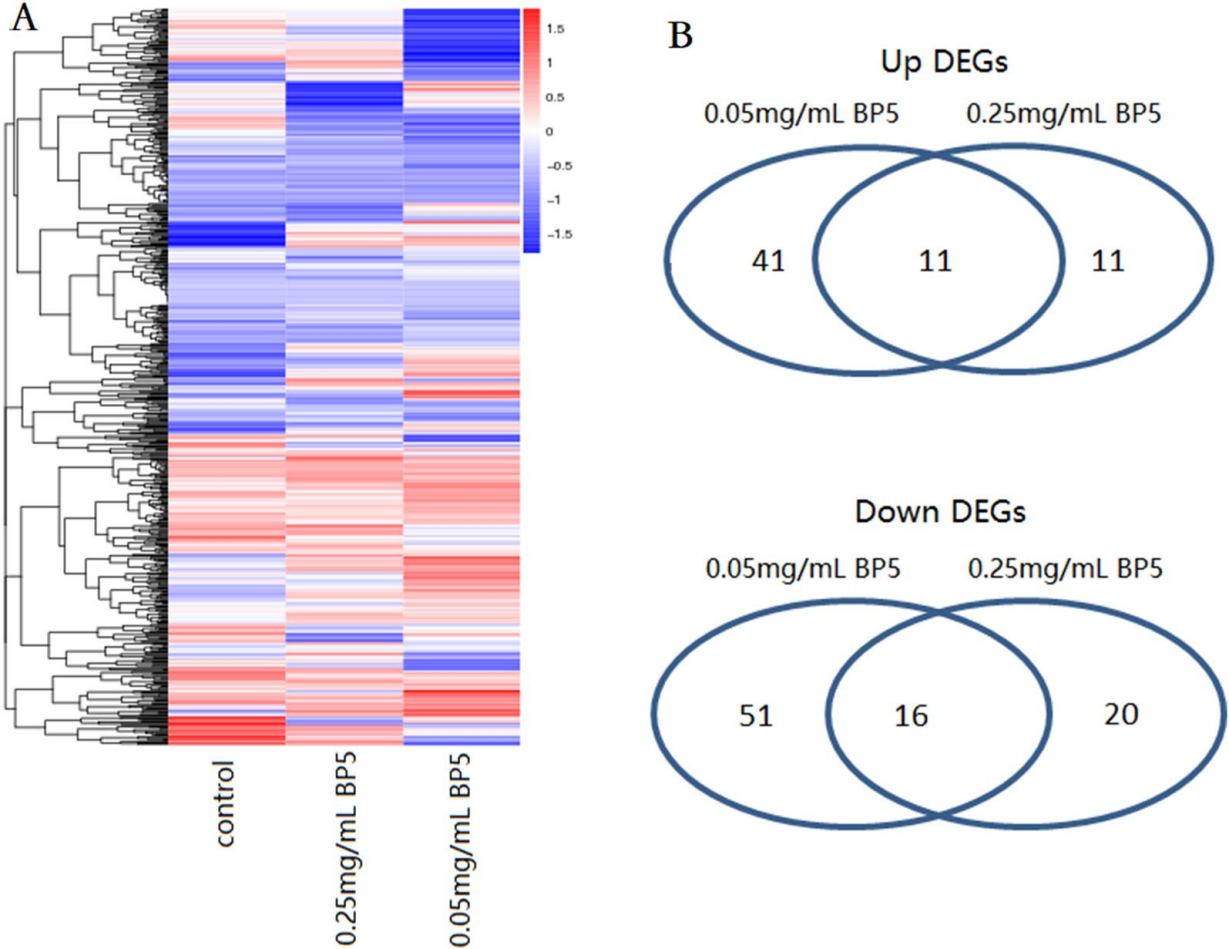
**Transcriptional factor analysis of DEG in spleen B cells with 0.05 and 0.25 mg/mL BP5 immunization.** Heatmap and Venn diagram of transcriptional factor of expressed genes in B cells from mice spleen immunized with 0.05 mg/mL and 0.25 mg/mL BP5 immunization. **A** The heatmap of the transcriptional factor genes in B cells from mice spleen immunized with or without 0.05 mg/mL and 0.25 mg/mL BP5 immunization. **B** Venn diagram showing the number of DEG of transcriptional factors between 0.05 and 0.25 mg/mL BP5 immunization. The numbers of down-regulated and up-regulated DEG in both comparisons were shown.

To explore the transcriptional factors in B cells from mice immunized with BP5 stimulation, their differential expression was analyzed. The heat maps shown in Figure 6A illustrate the overall expression profiles of B cells from the control, 0.05 and 0.25 mg/mL BP5 groups, respectively.

The results of differentially expressed transcription gene factors show that following 0.05 mg/mL BP5 immunization, there were 119 DEG, including 52 upregulated and 67 downregulated genes in B cells from the immunized mice (Figure 6B). Upon 0.25 mg/mL BP5 immunization, there were 58 DEG, including 22 upregulated and 36 downregulated genes in B cells from the immunized mice (Figure 6B). There were 41 specifically upregulated genes and 51 specifically downregulated genes following 0.05 mg/mL BP5 immunization, and 11 specifically upregulated genes and 20 specifically downregulated genes upon 0.25 mg/mL BP5 immunization. It was observed that there were 11 upregulated and 16 downregulated common genes in both B cells from mice immunized with 0.05 and 0.25 mg/mL BP5, in which upregulated transcription factor genes mainly included zf-C2H2, MYB, SAND, ZBTB and ETS families, and downregulated transcription factor genes mainly included bZIP, Homeobox, and seven other families (Table 4).

**Table 4:** **Common regulated transcription factor genes in response to 0.05 and 0.25 mg/mL BP5 immunization**

AGI code	Family	Fold-change	
		0.05 mg/mL BP5	0.25 mg/mL BP5
Up DEGs			
ENSMUSG00000032425	zf-C2H2	2.469929669	2.55405662
ENSMUSG00000089857	zf-C2H2	1.150712737	1.907080314
ENSMUSG00000092260	zf-C2H2	1.188187443	1.137200098
ENSMUSG00000074731	zf-C2H2	4.146608339	4.992810188
ENSMUSG00000025912	MYB	2.216510352	1.628045896
ENSMUSG00000005045	MYB	1.784038259	1.931409644
ENSMUSG00000090186	SAND	1.288627344	1.186491313
ENSMUSG00000099693	SAND	1.447989056	1.340733492
ENSMUSG00000071661	ZBTB	1.561645838	1.440269165
ENSMUSG00000030677	ZBTB	1.193712574	1.011425866
ENSMUSG00000012350	ETS	1.239717743	1.085919593
Down DEGs			
ENSMUSG00000029135	TF_bZIP	−1.30294048	−1.09451144
ENSMUSG00000030149	TF_bZIP	−1.41326318	−1.04566396
ENSMUSG00000033027	TF_bZIP	−1.90595971	−1.08247794
ENSMUSG00000050241	TF_bZIP	−1.6410691	−1.02349162
ENSMUSG00000027938	TF_bZIP	−4.19324166	−4.17711481
ENSMUSG00000038872	Homeobox	−4.1107795	−2.09465265
ENSMUSG00000030789	Homeobox	−1.85471706	−1.13597612
ENSMUSG00000040289	bHLH	−2.23277003	−1.63168068
ENSMUSG00000049807	bHLH	−1.85981793	−3.0660835
ENSMUSG00000044676	zf-C2H2	−1.36211858	−1.34599173
ENSMUSG00000022500	zf-LITAF-like	−1.14269906	−1.17288492
ENSMUSG00000095134	zf-MIZ	−1.45073789	−2.20512919
ENSMUSG00000028341	NGFIB-like	−1.09370599	−1.42636273
ENSMUSG00000032446	T-box	−1.32930321	−1.09305055
ENSMUSG00000100763	HMG	−3.02331666	−4.00718981
ENSMUSG00000032238	THR-like	−2.34524476	−1.00718981

## Discussion

BF is the vital central immune organ unique to birds and plays a key role in the differentiation and maturation of B cells [23]. BP5 is reported to increase the antibody production and induce B cell development [15, 24]. However, the function and mechanism of BP5 on B cell differentiation is still unknown. The exploration of the immune function and functional mechanism of bioactive peptide from BF on B cell development will provide insight into the mainstream immune induction and signaling pathways activated for the specific antigen stimulation.

During immune response, B cells need to be stimulated by antigens to be activated [25]. In this study, the activated B cells from mice immunized with AIV vaccine were selected as a B cell model. In this paper, it was observed that treatment with BP5 in vitro promoted total B cells, activated B cells, differentiated B cells, mature B cells and plasma cells, but also BP5 in vivo stimulated activated B cells and differentiated B cells. The different subsets of B and plasma cells have been associated with the humoural immune responses [26, 27]. These results suggest that BP5 might participate in the regulation of multiple stages of B cell development, and the regulation of BP5 on B cell differentiation might be different between in vitro stimulation and in vivo immunization, and the actions of BP5 did not completely depend on the microenvironment in BF.

B cells are the acknowledged critical mediators of the humoral immunity, and B cell differentiation is controlled by a variety of mRNA [28]. In order to investigate a deeper understanding of the molecular mechanism of bursal-derived active peptides on the regulation of B cell differentiation, in this paper, the spleen B cells from mice immunized with AIV vaccine plus 0.05 or 0.25 mg/mL BP5 were isolated and purified to detect the gene expression profiles of B cells with RNA sequencing technology. The results of DEG show that 471 genes were up-regulated and 657 genes were down in spleen B cells from the 0.05 mg/mL BP5 immunization group, and 185 genes were up-regulated and 286 genes were down-regulated in 0.25 mg/mL BP5 immunization group. These results suggest that BP5 could induce the differential expression of numerous genes in spleen B cells, in which the relationship of these DEG with B cell differentiation and maturation need to be further explored.

GO analysis is a systematic annotation for the functional properties of gene products, and can be used to facilitate the computational prediction of gene function [29]. In this study, we observed that BP5 at 0.05 and 0.25 mg/mL concentrations induced various biological functions and cellular activities, including metabolic processes and catalytic activity. It was reported that in addition to meeting the unique metabolic demands for B cell differentiation stages [30, 31], metabolites and tissue-specific signals could influence B cell fate [32, 33]. These results suggest that bursal-derived BP5 might activate various metabolic programs, receptors and co-activation molecules, leading to B lymphocyte maturation and functional orientation.

To further investigate the molecular basis of BP5 on B cell differentiation, the DEG involved KEGG pathways were analyzed. It was found that the DEG in B cells from mice immunized with 0.05 and 0.25 mg/mL BP5 were involved in five enrichment pathways, in which natural killer cell mediated cytotoxicity was one of the common enrichment pathways. Natural killer (NK) cells were the major lymphocyte subset of the innate immune system [34], which could play various roles during immune response, including exhibiting antibody-dependent cell cytotoxicity [35]. It was observed that seven downregulated genes were involved in natural killer cell mediated cytotoxicity in spleen B cells with both 0.05 mg/mL and 0.25 mg/mL BP5 immunizations. Also, there were seven natural killer cell related significant enriched biological processes in spleen B cells with BP5 immunizations at both concentrations. These results imply that BP5 might induce various biological functions related to NK cells, including the mechanism of genes shared with NK cells that were induced with BP5 stimulation on B cell development, however, this remains to be further explored.

Cytokine-cytokine receptor interaction was another significant enrichment pathway in spleen B cells with 0.05 and 0.25 mg/mL BP5 immunization, in which nine gene expressions were decreased. The cytokine microenvironment, and the intracellular and extracellular metabolic signals play a pivotal role in controlling the balance between regulatory and antibody-producing B cell subsets [32]. Cytokines are a vital component in the host immune system, and cytokine-receptor cross-reactivity and related signaling pathways are considered to be the primary drivers of cytokine pleiotropy [36]. Furthermore, various DEG were observed to participate in nine cytokine related enriched biological processes, including interferon-gamma, interleukin-18 and interleukin-6 cytokine production and the chemokine biosynthetic process. These results imply that cytokine mediated biological functions might be involved in the differentiation and maturation of B cells stimulated by BP5.

Antigen processing and presentation is a fundamental pathway in vertebrates, in which the epitope peptides forming the intracellular proteome interact with MHC-I or MHC-II, to enable different aspects of adaptive immunity to emerge [37, 38]. In this paper, 0.05 and 0.25 mg/mL BP5 immunization induced various DEG involved in antigen processing and presentation pathways, and also involved in antigen receptor-mediated signaling pathways and MHC class Ib receptor activity. Additionally, we found that various common DEG in both 0.05 and 0.25 mg/mL BP5 immunization were involved in B cell and immunoglobulin related biological processes. These results suggest that BP5 might regulate antigen processing and presentation during vaccine immunization, resulting in B cell differentiation and the humoral immune responses.

Furthermore, the hematopoietic cells in bone marrow are multipotent progenitors that involve various stages of the B-cell lineage [39]. BP5 at two experimental dosages regulated the expression of five genes involved in hematopoietic cell lineage pathways in spleen B cells, suggesting that except for bone marrow, the development and maturation of B cells continued to occur in other immune organs during vaccination.

The innate immune response activation is an intricate network, in which various immune components, such as the complement system, coagulation cascade and natural antibodies are interconnected [40], in which the complex complement and coagulation cascades interact with each other [41]. In this paper, the three genes Plau, C3 and Itgax were downregulated in both 0.05 mg/mL and 0.25 mg/mL BP5 immunizations, which might inhibit the activation of complement and coagulation cascades to control natural antibody-mediated response.

It is reported that B cells represent one of two essential components of immune responses, in which the critical transcriptional factors are involved in the interaction between B cells and macrophages [42, 43]. Transcription factors could play a vital role in signal transduction and gene expression of downstream functional genes in B cells. In this paper, the expression levels of 27 transcription factor genes were significantly differentially expressed in spleen B cells from mice immunized with 0.05 mg/mL and 0.25 mg/mL BP5, including 11 upregulated and 16 downregulated genes. These upregulated transcription factor genes included zf-C2H2, MYB, SAND, ZBTB and ETS families, and downregulated transcription factor genes mainly included bZIP, Homeobox, and seven other families.

C2H2 and bZIP domains are found in various transcriptional factors. The B-cell CLL/lymphoma 11B (Bcl11b) is a C2H2 zinc finger transcriptional factor that negatively regulates GPC differentiation [44]. Also the GFI1 and GFI1B genes, which contain six c-terminal C2H2 zinc finger motifs, are critical for both the innate and acquired immune system [45]. The Epstein-Barr virus EBNA 3C gene, containing the bZIP domain, functions as a regulator of viral and cellular transcription, and plays the vital role on B cell immortalization and functions [46]. In this study, four transcription factor genes of the zf-C2H2 family were significantly upregulated, and five transcription factor genes of the bZIP were significantly downregulated. These results suggest that the C2H2 and bZIP families’ transcription factors might regulate B cell differentiation following BP5 treatment.

Additionally, the Myc family is reported to regulate B cell growth and proliferation [47], which is related to the activated BCR signaling the differentiation of B cells [48, 49]. The ZBTB family is reported to be involved in the development, differentiation, and function of B cells [50]. Also, the homeobox NKX2-3 has been shown to activate B-cell receptor signaling and shape lymphocyte dynamics [51]. B-1b cell was reported to possess unique bHLH-driven p62-dependent self-renewal and atheroprotection [52]. In this paper, two MYB and two ZBTB transcriptional factors were significantly upregulated, and two homeobox and two bHLH transcriptional factors were significantly downregulated. In addition, single genes of various transcription factor families were downregulated both in 0.05 and 0.25 mg/mL BP5 treatment. These results suggest that BP5 might induce various transcriptional factor families that modulate the innate and acquired immune responses, and these transcript factor networks could also participate in B cell differentiation and maturation.

In general, BF is critical for B cell development and differentiation in birds. BP5, the active peptide derived bursa, induced the increased proportion of multiple B cell subtypes in vitro. Also, BP5 stimulated the activation and differentiation of B cells in vivo. Furthermore, the results of RNA sequencing technology show that BP5 regulated various DEG and transcriptional factors in spleen B cells, which were predicted to be involved in regulation of B cell development and differentiation. These results provide the basis to further evaluate the mechanisms behind BP5 induced B cell differentiation, and also might provide an important experimental basis for improving vaccine immunity.

## Supplementary Information

**Additional file 1.** The primers for qPCR.

**Additional file 2.** Antibody levels and spleen cell viabilities. **A** Antibody levels with ELISA. **B** HI antibody levels. **C** Flow cytometry plot of B lymphocytes purified with magnetic beads from the spleen cells of the immunized mice. **D** Spleen cell viabilities. Data represent the mean ± S.D. Significant differences between groups were determined using the student *t*-test. *P < 0.05, **P < 0.01.

**Additional file 3.** Mapping region among three experimental groups.

**Additional file 4.** Common Natural killer cell related biological processes in response to 0.05 and 0.25 mg/mL BP5 immunization.

**Additional file 5.** Cytokine related biological processes in response to 0.05 and 0.25 mg/mL BP5 immunization.

**Additional file 6.** Antigen and B cell related biological processes in response to 0.05 and 0.25 mg/mL BP5 immunization.

**Additional file 7.** Significant pathway enrichment in response to 0.05 mg/mL BP5 immunization.

**Additional file 8.** Significant pathways enrichment in response to 0.25 mg/mL BP5 immunization.

## Data Availability

The study was conducted in accordance with the recommendations in the Guidelines on Ethical Treatment of Experimental Animal" (2006) No. 398 published by the Ministry of Science and Technology, China and the Regulation regarding the Management" published by the Jiangsu Provincial People's Government, and approved by Animal Ethics Committee at Nanjing Agricultural University (Protocol No. PZ2019011, Feb 23, 2019).
