# Supplementary material for: Transcriptomic analysis of spleen B cell revealed the molecular basis of bursopentin on B cell differentiation
Source: Vet Res. 2022 Dec 14;53:109. doi: 10.1186/s13567-022-01123-z (PMC9753308; doi:10.1186/s13567-022-01123-z)
Supplement: Supplementary file 1 — Additional file 1. The primers for qPCR. [file 13567_2022_1123_MOESM1_ESM.docx]

**Additional file 1. The primers for qPCR**.

| Gene | F-primer | R-primer |
| --- | --- | --- |
| BC018473 | GACTCCTAGCCACGAGATGC | GACTCCTAGCCACGAGATGC |
| St6galnac1 | AGCCTCGGTGGGATTTTGAG | CTAGACGGTTCCACTCGCTC |
| Tmod2 | AAGGGTCCTGTCTCGGAGTG | CTTAGAGCCAGGAGTGAGCG |
| Bmp8b | CCCTCTTGGTAGCCTCTCCA | TGTGCGCCAAGCTATTCTCT |
| Tnfrsf21 | CTGGGGTGTGCGGAAGAAAG | ACCACCTCCAGGTTCTGACC |
| Csf1r | GTGACCCTGCGATGTGTGAG | TTCCGGGAGATTCAGGGTCC |
| Ackr3 | CATGAGCGTGGACCGCTATC | GCATACAACACGGCGTACCA |
| β-actin | TCTGGCACCACACCTTCTAC | ATCTGGGTCATCTTCTCGC |
