# Supplementary figures and images for: Transcriptomic analysis of spleen B cell revealed the molecular basis of bursopentin on B cell differentiation
Source: Vet Res. 2022 Dec 14;53:109. doi: 10.1186/s13567-022-01123-z (PMC9753308; doi:10.1186/s13567-022-01123-z)

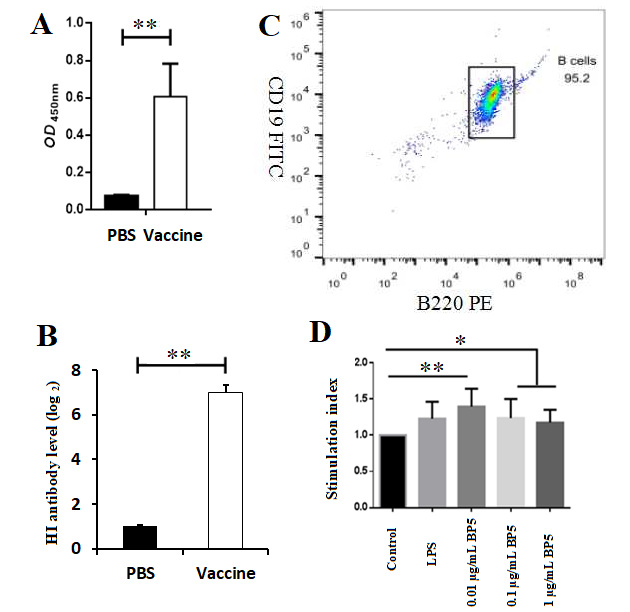

Supplement: Supplementary file 2 — Additional file 2. Antibody levels and spleen cell viabilities. A Antibody levels with ELISA. B HI antibody levels. C Flow cytometry plot of B lymphocytes purified with magnetic beads from the spleen cells of the immunized mice. D Spleen cell viabilities. Data represent the mean ± S.D. Significant differences between groups were determined using the student t-test. *P < 0.05, **P < 0.01. [file 13567_2022_1123_MOESM2_ESM.tif]
