# Supplementary material for: Transcriptomic analysis of spleen B cell revealed the molecular basis of bursopentin on B cell differentiation
Source: Vet Res. 2022 Dec 14;53:109. doi: 10.1186/s13567-022-01123-z (PMC9753308; doi:10.1186/s13567-022-01123-z)
Supplement: Supplementary file 3 — Additional file 3. Mapping region among three experimental groups. [file 13567_2022_1123_MOESM3_ESM.docx]

**Additional file 3. Mapping region among three experimental groups**.

| Library | Control | 0.05 mg/mL BP5 | 0.25 mg/mL BP5 |
| --- | --- | --- | --- |
| Exon | 16 701 221 (72.56%) | 17 287 673 (75.38%) | 16 714 171 (73.36%) |
| Intron | 5 235 909 (22.75%) | 4 492 231 (19.59%) | 5 012 396 (22.00%) |
| Intergenic | 1 078 582 (4.69%) | 1 154 980 (5.04%) | 1 057 191 (4.64%) |
