# Supplementary material for: Transcriptomic analysis of spleen B cell revealed the molecular basis of bursopentin on B cell differentiation
Source: Vet Res. 2022 Dec 14;53:109. doi: 10.1186/s13567-022-01123-z (PMC9753308; doi:10.1186/s13567-022-01123-z)
Supplement: Supplementary file 4 — Additional file 4. Common Natural killer cell related biological processes in response to 0.05 and 0.25 mg/mL BP5 immunization. [file 13567_2022_1123_MOESM4_ESM.docx]

**Additional file 4. Common Natural killer cell related biological processes in response to 0.05 and 0.25 mg/mL BP5 immunization**.

|  |  | FDR | | Up DEGs | | Down DEGs | |
| --- | --- | --- | --- | --- | --- | --- | --- |
| Accession | Term_name | 0.05 mg/mL BP5 | 0.25 mg/mL BP5 | 0.05 mg/mL BP5 | 0.25 mg/mL BP5 | 0.05 mg/mL BP5 | 0.25 mg/mL BP5 |
| GO:2000501 | regulation of natural killer cell chemotaxis | 0.000995955 | 0.000108492 | 0 | 0 | 4 | 4 |
| GO:0042269 | regulation of natural killer cell mediated cytotoxicity | 0.018658576 | 0.000361427 | 1 | 0 | 7 | 8 |
| GO:0002715 | regulation of natural killer cell mediated immunity | 0.019656756 | 0.000379803 | 1 | 0 | 7 | 8 |
| GO:2000502 | negative regulation of natural killer cell chemotaxis | 0.025152247 | 0.013350855 | 0 | 0 | 2 | 2 |
| GO:0045954 | positive regulation of natural killer cell mediated cytotoxicity | 0.032067635 | 0.000329938 | 0 | 0 | 5 | 6 |
| GO:0002717 | positive regulation of natural killer cell mediated immunity | 0.035405599 | 0.000370515 | 0 | 0 | 5 | 6 |
| GO:0002228 | natural killer cell mediated immunity | 0.038680533 | 0.007001551 | 0 | 0 | 4 | 4 |
