# Supplementary material for: Transcriptomic analysis of spleen B cell revealed the molecular basis of bursopentin on B cell differentiation
Source: Vet Res. 2022 Dec 14;53:109. doi: 10.1186/s13567-022-01123-z (PMC9753308; doi:10.1186/s13567-022-01123-z)
Supplement: Supplementary file 5 — Additional file 5. Cytokine related biological processes in response to 0.05 and 0.25 mg/mL BP5 immunization. [file 13567_2022_1123_MOESM5_ESM.docx]

**Additional file 5. Cytokine related biological processes in response to 0.05 and 0.25mg/mL BP5 immunization**.

| Accession | Term_name | FDR | | Up DEGs | | Down DEGs | |
| --- | --- | --- | --- | --- | --- | --- | --- |
|  |  | 0.05 mg/mL BP5 | 0.25 mg/mL BP5 | 0.05 mg/mL BP5 | 0.25 mg/mL BP5 | 0.05 mg/mL BP5 | 0.25 mg/mL BP5 |
| GO:0001816 | cytokine production | 3.69E-05 | 0.0147652 | 1 | 1 | 14 | 6 |
| GO:0045080 | positive regulation of chemokine biosynthetic process | 0.0003396 | 0.0005251 | 1 | 1 | 4 | 3 |
| GO:0071351 | cellular response to interleukin-18 | 0.0006761 | 0.0133509 | 0 | 0 | 3 | 2 |
| GO:0045073 | regulation of chemokine biosynthetic process | 0.0009738 | 0.0012119 | 1 | 1 | 4 | 3 |
| GO:0032649 | regulation of interferon-gamma production | 0.0051457 | 0.0070016 | 1 | 1 | 11 | 7 |
| GO:0070673 | response to interleukin-18 | 0.0069215 | 0.0380181 | 0 | 0 | 3 | 2 |
| GO:0032609 | interferon-gamma production | 0.0094547 | 0.0171303 | 0 | 0 | 4 | 3 |
| GO:0045410 | positive regulation of interleukin-6 biosynthetic process | 0.0112332 | 0.0200112 | 0 | 0 | 4 | 3 |
