# Supplementary material for: Transcriptomic analysis of spleen B cell revealed the molecular basis of bursopentin on B cell differentiation
Source: Vet Res. 2022 Dec 14;53:109. doi: 10.1186/s13567-022-01123-z (PMC9753308; doi:10.1186/s13567-022-01123-z)
Supplement: Supplementary file 6 — Additional file 6. Antigen and B cell related biological processes in response to 0.05 and 0.25 mg/mL BP5 immunization. [file 13567_2022_1123_MOESM6_ESM.docx]

**Additional file 6. Antigen and B cell related biological processes in response to 0.05 and 0.25mg/mL BP5 immunization**.

| Accession | Term_name | FDR | | Up DEGs | | Down DEGs | |
| --- | --- | --- | --- | --- | --- | --- | --- |
|  |  | 0.05 mg/mL BP5 | 0.25 mg/mL BP5 | 0.05 mg/mL BP5 | 0.25 mg/mL BP5 | 0.05 mg/mL BP5 | 0.25 mg/mL BP5 |
| GO:0050851 | antigen receptor-mediated signaling pathway | 0.0359149 | 4.052E-06 | 5 | 11 | 15 | 9 |
| GO:0032394 | MHC class Ib receptor activity | 0.0233995 | 0.0151261 | 0 | 0 | 2 | 2 |
| GO:0003823 | antigen binding | 0.0351444 | 3.499E-06 | 4 | 16 | 22 | 8 |
| GO:0002714 | positive regulation of B cell mediated immunity | 0.00373 | 0.0171303 | 0 | 1 | 8 | 4 |
| GO:0002712 | regulation of B cell mediated immunity | 0.0116166 | 0.0349521 | 0 | 1 | 8 | 4 |
| GO:0002891 | positive regulation of immunoglobulin mediated immune response | 0.00373 | 0.0171303 | 0 | 1 | 8 | 4 |
| GO:0002889 | regulation of immunoglobulin mediated immune response | 0.0116166 | 0.0349521 | 0 | 1 | 8 | 4 |
