# Supplementary material for: Transcriptomic analysis of spleen B cell revealed the molecular basis of bursopentin on B cell differentiation
Source: Vet Res. 2022 Dec 14;53:109. doi: 10.1186/s13567-022-01123-z (PMC9753308; doi:10.1186/s13567-022-01123-z)
Supplement: Supplementary file 7 — Additional file 7. Significant pathway enrichment in response to 0.05 mg/mL BP5 immunization. [file 13567_2022_1123_MOESM7_ESM.docx]

**Additional file 7. Significant pathways enrichment in response to 0.05 mg/mL BP5 immunization**.

| Name | Map | q | Up DEGs | Down DEGs |
| --- | --- | --- | --- | --- |
| IL-17 signaling pathway | map04657 | 0.000895 | 1 | 14 |
| C-type lectin receptor signaling pathway | map04625 | 0.001549 | 3 | 10 |
| Chemokine signaling pathway | map04062 | 0.005928 | 3 | 13 |
| Osteoclast differentiation | map04380 | 0.008257 | 3 | 9 |
| TNF signaling pathway | map04668 | 0.008257 | 2 | 9 |
| Toll-like receptor signaling pathway | map04620 | 0.01143 | 5 | 5 |
| Primary immunodeficiency | map05340 | 0.031738 | 1 | 4 |
| Th1 and Th2 cell differentiation | map04658 | 0.031767 | 0 | 8 |
| NF-kappa B signaling pathway | map04064 | 0.038574 | 1 | 8 |
| Rap1 signaling pathway | map04015 | 0.040296 | 3 | 13 |
| T cell receptor signaling pathway | map04660 | 0.040296 | 1 | 9 |
| Apoptosis | map04210 | 0.003635 | 7 | 10 |
| Adherens junction | map04520 | 0.004678 | 1 | 11 |
