# Supplementary material for: Transcriptomic analysis of spleen B cell revealed the molecular basis of bursopentin on B cell differentiation
Source: Vet Res. 2022 Dec 14;53:109. doi: 10.1186/s13567-022-01123-z (PMC9753308; doi:10.1186/s13567-022-01123-z)
Supplement: Supplementary file 8 — Additional file 8. Significant pathways enrichment in response to 0.25 mg/mL BP5 immunization. [file 13567_2022_1123_MOESM8_ESM.docx]

**Additional file 8. Significant pathways enrichment in response to 0.25 mg/mL BP5 immunization**.

| Name | Map | q | Up DEGs | Down DEGs |
| --- | --- | --- | --- | --- |
| Th17 cell differentiation | map04659 | 0.022846 | 2 | 5 |
| Aldosterone synthesis and secretion | map04925 | 0.039702 | 1 | 5 |
| Thyroid hormone synthesis | map04918 | 0.042395 | 1 | 4 |
